# Post-Traumatic Middle Cerebral Artery Pseudoaneurysm Following Depressed Skull Fracture: Case Report and PRISMA-Guided Literature Review

**DOI:** 10.3390/neurosci7040078

**Published:** 2026-07-08

**Authors:** Giuseppina Bevacqua, Eleonora Becattini, Valentina Grespi, Carlo Conti

**Affiliations:** 1 Department of Medicine and Surgery, University of Perugia, 06123 Perugia, Italy; 2 Neurosurgery Unit, Department of Neuroscience, Santa Maria Hospital, 05100 Terni, Italy; e.becattini@aospterni.it (E.B.); c.conti@aospterni.it (C.C.); 3 Stem Cell Laboratory, Cell Factory and Biobank, Santa Maria Hospital, 05100 Terni, Italy; v.grespi@aospterni.it

**Keywords:** pseudoaneurysms, traumatic intracranial aneurysms, head injury

## Abstract

Traumatic intracranial aneurysms (TICAs) are rare lesions accounting for <1% of intracranial aneurysms and are associated with high morbidity and mortality. They frequently arise after blunt trauma and may develop days to months following the initial injury, often evading early vascular imaging. Pseudoaneurysms predominate and carry a high risk of rupture. We report the case of a patient who sustained a severe head injury with a comminuted open depressed temporoparietal skull fracture, epidural hematoma, subarachnoid hemorrhage, and mandibular fracture after a road traffic accident. Initial vascular imaging revealed no aneurysm. At six-month follow-up, CT angiography demonstrated a 7 × 5 mm pseudoaneurysm arising from the rolandic branch of the right M3 middle cerebral artery, within an area of post-traumatic encephalomalacia. The aneurysm was confirmed by MRI/MRA and treated successfully with microsurgical clipping. This case underscores the importance of delayed vascular imaging in trauma patients with skull fractures or parenchymal injury, even when early angiography is normal. TICAs may develop over several months, and timely identification permits definitive management before rupture.

## 1. Introduction

Traumatic intracranial aneurysms (TICAs) are uncommon vascular lesions representing less than 1% of all intracranial aneurysms. Although rare, they are characterized by an aggressive clinical behavior and a high risk of rupture [1,2]. The pathophysiology underlying TICA formation is not fully understood, and the heterogeneity of clinical presentations strongly suggests the existence of different subentities. Pseudoaneurysms constitute the most frequent histological subtype and arise from disruption of all layers of the arterial wall, with subsequent containment by surrounding tissues [3]. Owing to the absence of a true vessel wall, these lesions are inherently unstable and prone to unpredictable enlargement or rupture. Conservative management has been associated with mortality rates of up to 50% [4], whereas early diagnosis and prompt treatment are correlated with markedly improved outcomes and low mortality [5]. Despite this, timely intervention is not always feasible. One of the major diagnostic challenges is that TICAs may not be detectable on initial vascular imaging, as they can develop at any time from days to several months after the initial traumatic event. Early angiographic studies may therefore be falsely negative.

The literature reports numerous cases of delayed TICA formation, often diagnosed only after secondary hemorrhagic complications that might have been preventable with earlier detection and treatment. At present, there is no clear consensus regarding the optimal timing or the most appropriate imaging modality for screening patients with traumatic brain injury (TBI) who are at risk of developing delayed TICAs. This uncertainty poses a significant clinical dilemma, particularly in patients with skull fractures or parenchymal injuries located along the course of major intracranial vessels. Most reported TICAs involve the anterior circulation [6]; however, traumatic pseudoaneurysms of distal middle cerebral artery branches are particularly rare. We present a case of a delayed post-traumatic pseudoaneurysm of the M3 rolandic branch following a depressed skull fracture. In addition, we report our institutional experience with delayed TICAs and provide a systematic review of the literature, with particular attention to the interval between trauma and aneurysm diagnosis and/or rupture. The aim of this study is twofold: first, to report a rare case of delayed post-traumatic pseudoaneurysm arising from a distal M3 branch of the middle cerebral artery following a depressed skull fracture; second, to provide a focused review of the literature on traumatic intracranial aneurysms, with particular emphasis on mechanisms of delayed formation, timing of diagnosis, and the role of repeat vascular imaging in patients at risk. Through this combined approach, we aim to highlight the potential for delayed vascular complications even after initially negative imaging, and to support the need for tailored radiological follow-up in selected patients.

## 2. Case Presentation

A 17-year-old male was admitted on 17 July 2024, after a road traffic accident in which he was struck by a car. He was transported by emergency medical services to the Emergency Department of Perugia. On arrival, due to the severity of the head injury, the patient underwent orotracheal intubation and sedation (GCS 8) and was subsequently admitted to the Intensive Care Unit. Initial cranial CT imaging revealed a comminuted, open, angulated, and depressed right temporoparietal skull fracture, associated with post-traumatic subarachnoid hemorrhage and a right temporoparietal epidural hematoma. Additional injuries included a displaced fracture of the right mandibular condyle. No vascular abnormalities were identified on the initial imaging studies (Figure 1). Because of the severity of the traumatic brain injury and the location of the hemorrhagic lesions adjacent to the depressed skull fracture, CT angiography (CTA) was performed at admission and demonstrated no aneurysm or other vascular abnormality. Although the hemorrhage adjacent to the fracture site raised concern for an underlying vascular injury, the negative initial CTA together with the delayed appearance of the lesion at follow-up was consistent with delayed traumatic pseudoaneurysm formation. On 18 July 2024, the patient underwent urgent neurosurgical intervention, consisting of debridement of the lacerated temporoparietal wound, evacuation of hemorrhagic components, and reconstruction of the cranial vault. Subsequently, on 24 July 2024, he underwent maxillofacial surgery with osteosynthesis of the right mandibular condyle.

Following these procedures, the patient was gradually weaned from mechanical ventilation. Neurological examination at awakening showed the patient to be awake, cooperative, and oriented, with preserved motor function in all four limbs, except for mild left upper limb weakness (MRC grade 4/5). Serial postoperative cranial CT scans demonstrated a favorable post-surgical course without new hemorrhagic complications. During the remainder of hospitalization, repeated physiatric evaluations documented progressive improvement in motor strength, particularly in the left upper limb. The patient was discharged in good general and neurological condition on 8 August 2024.

### 2.1. Delayed Aneurysm Detection

During routine radiological follow-up in January 2025, a CT angiography (CTA) of the brain revealed an unexpected 7 × 5 mm saccular aneurysmal dilation arising from the rolandic branch of the right M3 segment of the middle cerebral artery, located within an area of post-traumatic encephalomalacia/cicatricial change corresponding to the site of the previous intraparenchymal hemorrhage (Figure 2). The vascular lesion was further characterized and confirmed on brain MRI with contrast enhancement and MR angiography performed on 21 January 2025, which demonstrated features consistent with a post-traumatic pseudoaneurysm (Figure 3).

### 2.2. Preoperative Neurological Status

At the time of neurosurgical evaluation, the patient was neurologically intact. On examination, he was awake, alert, cooperative, and fully oriented, with fluent speech and intact comprehension. Motor examination showed normal strength in all four limbs, with no residual sensory or motor deficits. Cranial nerve examination was unremarkable, including normal ocular motility and absence of diplopia. Deep tendon reflexes were symmetrical and within normal limits. The patient denied headache, visual disturbances, or other focal neurological symptoms, and gait was normal. Sphincter function was preserved.

### 2.3. Treatment and Outcome

Following multidisciplinary discussion and detailed counseling of the patient and his family regarding the available therapeutic strategies, elective microsurgical treatment with aneurysm clipping was proposed and accepted. The patient was admitted for definitive management. A right-sided microsurgical craniotomy was performed through reopening of the previous surgical incision. After removal of the bone flap and cruciate dural opening, microsurgical dissection was carried out under neuronavigation guidance. A dilated cortical sulcus surrounded by post-traumatic malacic brain tissue was identified. Careful microsurgical dissection allowed identification of a saccular dilation arising from a cortical arterial branch, consistent with a traumatic pseudoaneurysm. Both the afferent and efferent arterial segments were isolated, and definitive exclusion of the aneurysmal sac was achieved by application of a curved aneurysm clip while preserving parent vessel patency. Intraoperative indocyanine green videoangiography and micro-Doppler ultrasonography confirmed complete aneurysm exclusion with preserved distal flow. Duraplasty was performed using Tisseel sealant, the bone flap was repositioned and fixed with titanium miniplates, and a submuscular drain was placed before layered wound closure.

Postoperative CT and CTA confirmed complete obliteration of the pseudoaneurysm with preserved distal flow (Figure 4). The postoperative course was uneventful, and the patient was mobilized progressively. He was discharged in stable neurological condition, without new deficits.

## 3. Literature Review

A systematic literature search was conducted in accordance with PRISMA guidelines. The following electronic databases were queried: PubMed/MEDLINE, Scopus, and Web of Science, from database inception through October 2025. The search strategy combined Medical Subject Headings (MeSH) and free-text terms related to traumatic intracranial aneurysms and traumatic brain injury. The following keywords and Boolean operators were used:

(“intracranial aneurysm”[MeSH] OR “traumatic intracranial aneurysm” OR “traumatic cerebral aneurysm” OR “pseudoaneurysm” OR “cerebral pseudoaneurysm”)

AND (“brain injuries, traumatic”[MeSH] OR “traumatic brain injury” OR “head trauma” OR “head injury” OR TBI) AND (“delayed diagnosis” OR “time factors”[MeSH] OR “rupture” OR “outcome” OR “natural history”).

Relevant articles were identified through screening of published English-language literature. Reference lists of selected articles were also reviewed to identify additional eligible studies. Studies were included if they met the following criteria:

1. Reported patients older than 15 years. This cutoff was chosen to maintain a relatively homogeneous study population comparable to our index case and to avoid mixing pediatric and late-adolescent/adult traumatic intracranial aneurysms, which may differ in terms of injury mechanisms, vascular response, clinical management, and reporting standards.
2. Described traumatic intracranial aneurysms or pseudoaneurysms.
3. Provided available preoperative and postoperative clinical data.
4. Reported the type of treatment performed (microsurgical, endovascular, or combined).
5. Included clinical and/or radiological follow-up data.

Studies were excluded if they involved pediatric patients (≤15 years), lacked sufficient clinical detail, did not specify treatment modality, or provided no postoperative or follow-up information. From each eligible study, the following data were extracted: patient demographics, mechanism of injury, aneurysm location, time from trauma to diagnosis, imaging modality used for detection, treatment strategy, and clinical outcome.

A total of 132 records were identified through database searching (PubMed: 58; Scopus: 42; Web of Science: 32). After removal of 27 duplicates, 105 records were screened based on title and abstract. Of these, 61 were excluded as not relevant. The remaining 44 full-text articles were assessed for eligibility. After full-text review, 26 articles were excluded due to lack of relevant clinical data, pediatric population, or non-traumatic etiology.

Finally, 18 studies were included in the qualitative synthesis. Data were analyzed descriptively and synthesized qualitatively. Due to heterogeneity in study design and outcome reporting, no quantitative meta-analysis was performed. The results of the literature review were summarized in tabular form and compared with the findings of the present case (Table 1 and Table 2) [7,8,9,10,11,12,13,14,15,16,17,18,19,20,21,22,23,24,25,26,27,28,29,30,31,32,33,34,35,36,37].

## 4. Discussion

Traumatic intracranial aneurysms (TICAs) are rare entities, representing <1% of all intracranial aneurysms and showing a peculiar natural history characterized by fragility, a high risk of rupture, and unpredictable timing of presentation [38]. Although most TICAs arise in the anterior circulation [6], traumatic pseudoaneurysms of distal cortical branches—such as the M3 segment involved in our case—remain exceptionally uncommon and are often under-recognized due to their delayed and heterogeneous radiological appearance. The pathophysiological mechanisms underlying the formation of traumatic intracranial pseudoaneurysms are complex and often multifactorial. Proposed mechanisms include direct vessel wall injury caused by bone fragments in the setting of skull fractures, stretching or torsion of cortical vessels during the traumatic event, and progressive degeneration of the vascular wall due to contusion-related ischemia or necrosis.

In our case, the pseudoaneurysm developed within an area of post-traumatic encephalomalacia, suggesting a delayed process of vessel wall weakening. This observation is consistent with the concept that vascular injury may evolve over time rather than being immediately detectable after trauma. An iatrogenic vascular injury should always be considered in the differential diagnosis of delayed intracranial pseudoaneurysms, particularly in patients who have undergone previous neurosurgical procedures. However, in the present case, the delayed appearance of the lesion together with its close anatomical relationship to the depressed skull fracture and the surrounding post-traumatic parenchymal changes strongly favor a traumatic rather than an iatrogenic origin.

### 4.1. Delayed Diagnosis Is the Dominant Pattern

A consistent finding from our review is the lateness of diagnosis. Most pseudoaneurysms were identified after the first week, with some detected months after trauma [7,8,9,10,11,12,13,14,15,16,17,18,19,20,21,22,23,24,25,26,27,28,29,30,31,32,33,34,35,36,37,38]. This trend supports the theory that TICAs arise through progressive wall degradation rather than immediate vascular rupture. Nevertheless, a small but significant subset of cases demonstrates aneurysm formation within 24 h, highlighting that the temporal evolution of TICAs is heterogeneous and cannot be safely predicted on the basis of early imaging alone. Our case fits the more common pattern: an initially normal vascular study, followed by delayed aneurysm development identified on routine follow-up imaging. This aligns with reports showing that early negative CTA/DSA cannot exclude later pseudoaneurysm formation and underscores the need for scheduled vascular imaging in patients with skull fractures or parenchymal injury along major arterial trajectories [39].

### 4.2. Radiological Hallmarks and the Diagnostic Challenge

Pseudoaneurysms often exhibit irregular morphology, peripheral location, delayed filling and emptying, and a poorly defined or absent neck—features that were consistently described in the reviewed literature and matched the angiographic appearance of our case. From a diagnostic perspective, CT angiography (CTA) represents an excellent first-line imaging modality due to its rapid acquisition, wide availability, and non-invasive nature. However, CTA may not always be sufficient, particularly in the early phase following trauma, when vascular lesions may still be evolving. In such cases, a negative CTA does not exclude the presence or future development of a traumatic aneurysm. When clinical suspicion persists or when patients present with risk factors such as skull fractures, intracranial hemorrhage, or unexplained neurological deterioration, repeat vascular imaging should be considered. Digital subtraction angiography (DSA) remains the gold standard for diagnosis, particularly in equivocal cases or when non-invasive imaging is inconclusive. In our case, DSA was not initially performed because CTA and subsequent MRI/MRA provided sufficient anatomical characterization for surgical planning. Moreover, the lesion arose from a superficial cortical branch of the M3 segment rather than from a deep-seated vessel, allowing adequate visualization of the aneurysm morphology and its relationship with the parent artery using non-invasive vascular imaging alone.

### 4.3. Management Strategies and Outcomes

Our review demonstrated substantial variability in treatment strategies: parent vessel occlusion, trapping with or without bypass, direct clipping in selected cases [8,9,10,11,12,13,14,15,16,17,18,19,20,21,22,23,24,25,26,27,28,29,30,31,32,33,34,35,36], and endovascular coiling or glue embolization [2,3,4,5,6,7,8,9,10,11,12,13,14,15,16,17,18,19,20,21,22,23,24,25,26,27,28,29,30,31,32,33,34,35,36,37]. The choice depends on aneurysm morphology, vessel caliber, collateral circulation, and hemorrhagic risk [40,41,42]. Outcomes across the reviewed cases remain poor overall, with mortality rates up to 54% in untreated patients and only about one-third achieving a favorable Glasgow Outcome Score even after treatment [8]. In contrast, our patient achieved an excellent postoperative result following surgical clipping, likely due to a combination of favorable lesion accessibility (distal cortical location), preserved neurological function, and timely detection before rupture. Although direct microsurgical clipping was feasible in our patient because of the superficial cortical location of the lesion, alternative strategies may be preferable in other cases. Endovascular techniques, including stent-assisted coiling, flow-diverting stents, parent vessel occlusion, or coil embolization, are valuable options, particularly for deep-seated or surgically inaccessible lesions. Conversely, when parent vessel sacrifice is unavoidable and collateral circulation is insufficient, microsurgical revascularization with extracranial-to-intracranial bypass may be required to maintain distal cerebral perfusion. While routine vascular imaging in all traumatic brain injury cases is not justified, patients with specific risk factors—such as depressed skull fractures, subarachnoid hemorrhage, or focal contusions—may benefit from scheduled follow-up imaging to detect delayed vascular complications. These considerations highlight the importance of tailored radiological follow-up in selected patients. Our findings support a more cautious approach in these patients, with consideration of repeat imaging even in the presence of initially negative studies, particularly for posterior circulation TICAs, which have been associated with worse clinical outcomes.

## 5. Conclusions

Traumatic intracranial pseudoaneurysms are rare but potentially severe vascular complications of head trauma. One of the main diagnostic challenges is that these lesions may develop days to months after the initial injury and may not be visible on early vascular imaging. Our case, together with the reviewed literature, highlights the importance of maintaining clinical suspicion in patients with skull fractures, traumatic subarachnoid hemorrhage, cortical contusions, or parenchymal injuries located near major vascular structures.

In selected high-risk patients, repeat vascular imaging should be considered even after initially negative studies, particularly in the presence of delayed neurological changes or evolving radiological findings. CTA represents a practical and effective first-line imaging modality for follow-up evaluation, allowing timely identification of delayed vascular complications in most cases.

Early diagnosis and multidisciplinary management remain essential to reduce the risk of delayed rupture and improve clinical outcomes.

## Figures and Tables

**Figure 1:**
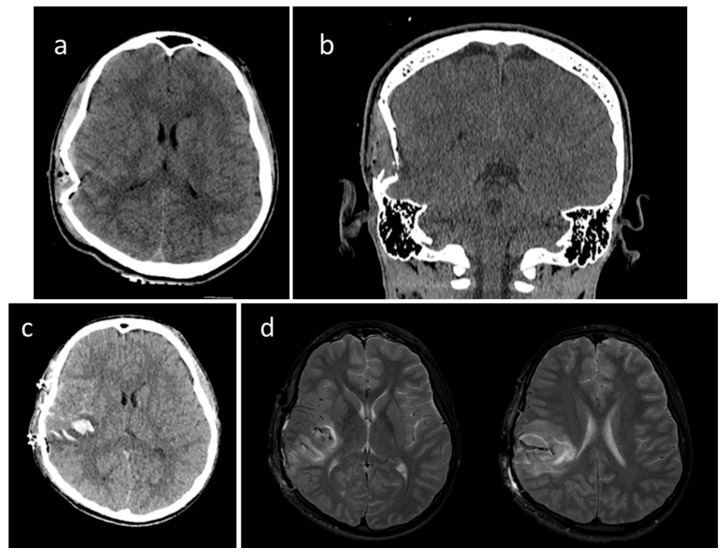
Preoperative cerebral CT (**a**) axial, (**b**) sagittal; (**c**) postoperative cerebral CT axial; (**d**) postoperative cerebral MR (axial T2).

**Figure 2:**
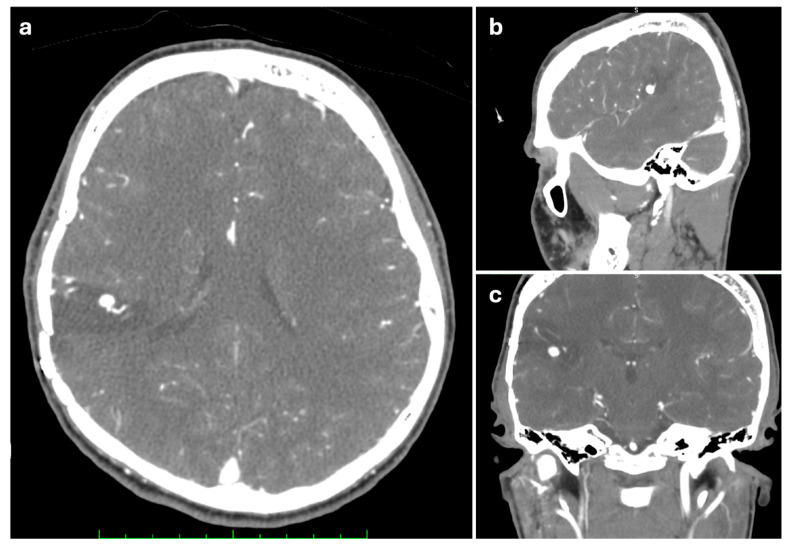
Cerebral CT angiography (CTA) showing a 7 × 5 mm saccular aneurysm of the rolandic branch of the right M3 segment of the middle cerebral artery, located within an area of post-traumatic encephalomalacia. (**a**) axial, (**b**) sagittal; (**c**) coronal.

**Figure 3:**
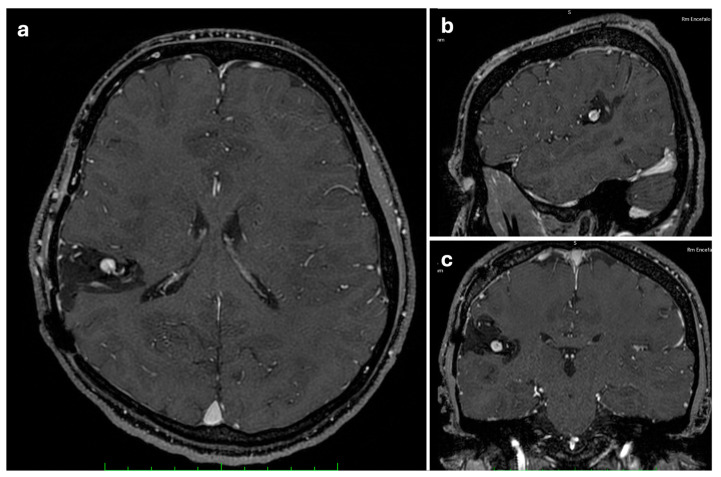
Cerebral MR angiography confirming the presence of a saccular aneurysm of the rolandic branch of the right M3 within a post-traumatic malacic area; (**a**) axial, (**b**) sagittal, (**c**) coronal.

**Figure 4:**
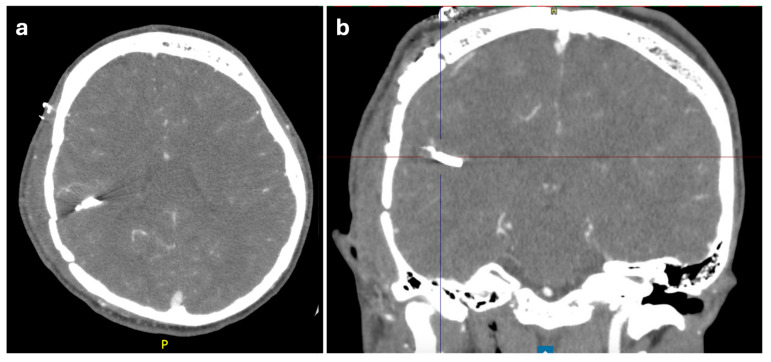
Postoperative CT and CTA showing the surgical clip and confirming the complete obliteration of the pseudoaneurysm; (**a**) axial; (**b**) coronal.

**Table 1:** Characteristics of traumatic intracranial aneurysms across studies (part 1).

	Authors	Year	N. Patients	Sex	Age	Mechanism of Injury	GCS	First CT
1	Hossain M. et al. [7]	2002	1	F	17	Fall from height	14	Depressed skull fracture, contusion
2	Nishioka [8]	2002	2	M	20	Head trauma	4	SAH, IVH
				F	33	Head trauma	14	SAH, minimal IVH
3	William W. et al. [9]	2005	1	M	34	Car accident	15	Jefferson’s fracture c1
4	Barua et al. [10]	2007	1	F	16	Gunshot	4	ICH
5	Mcelroy et al. [11]	2008	1	M	62	Head trauma	7	SAH, skull base fracture
6	Suhara S. et al. [12]	2008	1	F	27	Car accident	15	Negative
7	Binning MJ. et al. [13]	2009	1	M	15	Assault	7	SAH, IVH
8	Zanini MA. et al. [14]	2010	1	F	48	Craniotomy (clipping M1 aneurysm)	NA	SAH
9	Gaofeng XU et al. [15]	2011	1	M	27	Fall from height	3	SAH, contusion
10	Coulter [16]	2011	1	F	20	Car accident	15	SAH, left C1 lateral mass fracture, agf:pseudoaneurysm left VA at C0-C1 passage
11	Paiva Wellingson S. et al. [17]	2012	1	M	31	Assault	7	SAH, IVH with hydrocephalus, cerebellar contusion
12	Santos G. et al. [18]	2013	1	M	29	Gunshot	7	Contusion, ASDH, SAH, convexity and skull base fracture, pnumocephalus
13	Li T. et al. [19]	2013	1	M	34	Motorcycle accident	7	Skull base fracture, contusion
14	Sui M. et al. [20]	2015	1	M	54	Fall from height	15	Negative
15	Sun G. et al. [21]	2015	2	M	42	Car accident	9	ICH + SAH
				M	50	Car accident	7	ICH + SAH
16	Purgina B. et al. [22]	2015	1	M	22	Assault	3 (recover to 15 in ER)	SAH, IVH, hydrocephalus
17	Desouza [23]	2016	3	M	20	Assault	3 (recover to 15 in ER)	SAH, hydrocephalus
				M	21	Assault	14	SAH, hydrocephalus
				M	35	Assault	-	SAH, IVH, hydrocephalus
18	Lee [24]	2016	1	F	55	Head trauma	4	SAH, cerebellar ICH
19	Varma S. et al. [25]	2017	1	M	79	Fall from height	14	sADH
20	Policicchio D. et al. [26]	2018	1	M	19	Head trauma	15	ICH, skull base fracture, ocular trauma
21	Urszula M. et al. [27]	2020	1	M	30	Minor head trauma	15	Not performed
22	Niu Y. et al. [2]	2020	3	M	28	Head trauma	14	SAH, iVH
				M	38	Motorcycle accident	14	SAH
				M	18	Car accident	NA	Brain contusion, depressed skull fracture
23	Yeap M. et al. [28]	2021	1	M	59	Fall from height	15	ASDH
24	Palmieri M.et al. [29]	2022	1	M	26	Assault	8	Basal cistern and convexity SAH, IVH
25	Ikeuchi Y. et al. [30]	2022	1	F	69	Fall from height	11	SAH, IVH with hydrocephalus, contusion
26	Tsunoda S. et al. [31]	2022	1	M	81	Minor head trauma	14	ICH, SAH, bilateral ASDH
27	Nishihiro S. et al. [32]	2022	1	M	68	Fall from height	4	SAH, skull base fracture
28	Isozaki J. et al. [33]	2025	1	F	46	Car accident	6	Skull base fracture, pneumocephalus, SAH
29	Asad S. et al. [34]	2025	1	M	37	Assault	14	Extra-axial hemorrhage, IVH, SAH, skull convexity fracture
30	Watanabe T. et al. [35]	2025	1	F	20	Car accident	14	SAH, Lefort I and II fracture, wide-necked aneurysm in the cavernous portion of the right ICA
31	Chen K. et al. [36]	2025	1	F	34	Car accident	8	Contusions, SAH, skull convexity fracture
32	Sugiyama N. et al. [37]	2025	1	M	67	Falling	15	Skull base fracture

Abbreviations: SAH: subarachnoid hemorrhage; IVH: intraventricular hemorrhage; ICH: intracerebral hemorrhage; ADSH: acute sub-dural hematoma; AOD: atlanto-occipital dislocation; aSDH: subacute subdural hematoma; NA: not assessed.

**Table 2:** Characteristics of traumatic intracranial aneurysms across studies (part 2).

	Authors	Interval Trauma to Detection (Days)	Presentation	CT at Diagnosis	Location	Rupture (Yes/No)	Management	Outcome
1	Hossain M. et al. [7]	14	NA	IVH	Right PICA	Y	Endovascular	Paraplegic (burst fracture of T7, post-coil right arm weakness (MRC 3)
2	Nishioka [8]	11	Coma	Diagnosis with AGF at day 21 (no CT)	Right PICA	Y	None	Death
		14	Headache and stiff neck	Diagnosis with AGF at day 14 (no CT)	Left PICA	NA	Clipping	No deficit
3	William W. et al. [9]	60	Headache and stiff neck	Fracture of lateral mass and left foramen transversarium of C1	Left VA	N	Endovascular	No deficit
4	Barua et al. [10]	7	Coma	Left frontal ICH	Callosal marginal artery	Y	Clipping	NA
5	Mcelroy et al. [11]	21	NA	Negative	Tip of BA	N	Endovascular	NA
6	Suhara S. et al. [12]	30	Headache and seizure	ASDH	Perocallosal artery	Y	Clipping	No deficit
7	Binning MJ. et al. [13]	630	Negative	Negative	Left PICA	N	Endovascular	No deficit
8	Zanini MA. et al. [14]	180	Negative	Negative	M1 branch	N	Clipping	No deficit
9	Gaofeng XU et al. [15]	14	Negative	Focal SAH	(PCA) P3	Y	Endovascular	No deficit
10	Coulter [16]	0	NA	-	Left VA (C0-C1)	N	Endovascular	Good
11	Paiva Wellingson S. et al. [17]	0	Coma		SCA	Y	Endovascular	Coma
12	Santos G. et al. [18]	60	Negative	Left temporal hyperdense ovoid lesion	Right M2	Y	Clipping	No deficit
13	Li T. et al. [19]	33	Coma	Epistaxis	Midportion of BA	N	Endovascular	GCS 8 post op
14	Sui M. et al. [20]	6	Disturbance of consciousness and paralysis of the right limbs	Corpus callosum ICH	Left pericallosal artery	Y	Clipping	No deficit
15	Sun G. et al. [21]	3	GCS 6	Frontal lobe ICH	Pericallosal aneurysm	Y	Endovascular	Coma
		6	GCS 6	IVH	Pericallosal aneurysm	Y	Clipping	Good
16	Purgina B. et al. [22]	9	Loss of consciousness	IVH and SAH	Left PICA	Y	None	Death
17	Desouza [23]	11	Loss of consciousness	(post mortem diagnosis)	VA	Y	None	Death (post mortem diagnosis)
		0	Headache, drowsiness	-	Left PICA	Y	Endovascular	Good (recovering from emiparesis related to ICH after EVD removal)
		7	Loss of consciousness	Increased IVH	Left PICA	Y	Endovascular	Death
18	Lee [24]	30	GCS 15 (VI CN palsy)	Negative	Right PICA	N	Clipping	Good (recovering nerve palsy)
19	Varma S. et al. [25]	3	Weakness and expressive dysphasia	Increased sASDH	MCA (M3)	Y	Clipping	Good
20	Policicchio D. et al. [26]	180	Severe headache, vomiting and mild confusion	Left frontal ICH (DSA negative)		N	Clipping	No deficit
21	Urszula M. et al. [27]	16	GCS 3 with persistent ICP increase	Relapsing asdh	Left PCA (P3-P4)	Y	Endovascular	Cognitive impairment, right emiparesis
22	Niu Y. et al. [2]	16	Confusion	Negative	PICA	N	Endovascular	Good
		1	Headache		Posterior medial inferior frontal artery	Y	Endovascular	Good
		18	Fever and loss of consciousness	SAH	Right ICA	Y	Endovascular	Death
23	Yeap M. et al. [28]	19	ICH, GCS 7	Acute intracerebral hemorrhage	Left SCA	Y	Clipping	Afasia
24	Palmieri M.et al. [29]	9	GCS 3	Increased SAH and IVH	Left PICA	Y	Endovascular	Death
25	Ikeuchi Y. et al. [30]	0	GCS 4	Increased SAH, IVH and hydrocephalus	Right PICA (anterior medullary segment)	Y	Endovascular	Death
26	Tsunoda S. et al. [31]	0	NA		Bifurcation at the pericallosal segment of an azygos ACA	Y	Clipping	Secondary hydrocephalus, long-term care hospital
27	Nishihiro S. et al. [32]	1	GCS 4	Increased SAH and contusion	Left PICA	Y	Endovascular + pcf craniectomy	Poor
28	Isozaki J. et al. [33]	8	Coma		Petrous-to-cavernous junction of the right ICA	N	Endovascular	Poor
29	Asad S. et al. [34]	19	GCS 7	IVH and new onset hydrocephalus	Right PCA	Y	Endovascular	Death
30	Watanabe T. et al. [35]	0	III/IV nerve palsy		Right ICA	N	Endovascular	Good (partial III palsy in Lefort fracture)
31	Chen K. et al. [36]	21	Headache and persistent rhinorrhea	Right frontal ICH	Right ACA (A2)	Y	Clipping	Good
32	Sugiyama N. et al. [37]	3	GCS 4	Basal cistern SAH	Right PICA	Y	Endovascular	Poor (hypoglossal nerve palsy)

Abbreviations: SAH: subarachnoid hemorrhage; IVH: intraventricular hemorrhage; ICH: intracerebral hemorrhage; ADSH: acute sub-dural hematoma; AOD: atlanto-occipital dislocation; aSDH: subacute subdural hematoma; PICA: postero-inferior cerebellar artery; VA: vertebral artery; PCA: posterior cerebral artery; BA: basilar artery; SCA: superior cerebellar artery; MCA: middle cerebral artery; ICA: internal carotid artery; ACA: anterior cerebral artery; NA: not assessed.

## Data Availability

Data from the study will be available upon reasonable request to the corresponding author.

## Abbreviations

The following abbreviations are used in this manuscript:

TICA	Traumatic intracranial aneurysm
MCA	Middle cerebral artery
CTA	Cerebral computed tomography angiography
TBI	Traumatic brain injury
